# Evidence of *sp*^2^-like Hybridization of Silicon Valence Orbitals in Thin and Thick Si Grown on α-Phase Si(111)√3 × √3R30°-Bi

**DOI:** 10.3390/ma15051730

**Published:** 2022-02-25

**Authors:** David Garagnani, Paola De Padova, Carlo Ottaviani, Claudio Quaresima, Amanda Generosi, Barbara Paci, Bruno Olivieri, Mieczysław Jałochowski, Mariusz Krawiec

**Affiliations:** 1 Consiglio Nazionale delle Ricerche—ISM, Via Fosso del Cavaliere 100, 00133 Roma, Italy; davidgaragnani@gmail.com (D.G.); carlo.ottaviani@ism.cnr.it (C.O.); claudio.quaresima@ism.cnr.it (C.Q.); amanda.generosi@artov.ism.cnr.it (A.G.); barbara.paci@ism.cnr.it (B.P.); 2 Instituto Nazionale di Fisica Nucleare-Laboratori Nazionali di Frascati—INFN-LNF, Via Enrico Fermi 54, 00044 Frascati, Italy; 3 Consiglio Nazionale delle Ricerche—ISAC, Via Fosso del Cavaliere 100, 00133 Roma, Italy; brunoolivieri42@gmail.com; 4 Institute of Physics, Maria Curie-Sklodowska University, pl. M. Curie-Sklodowskiej 1, 20-031 Lublin, Poland; mieczyslaw.jalochowski@mail.umcs.pl

**Keywords:** silicene-like, α-phase Si(111)√3 × √3R30°-Bi, Si K-edge, *sp*^2^-like hybridization, reflection electron energy loss spectroscopy, Auger spectroscopy, low-energy electron diffraction, reflection high-energy electron diffraction, scanning tunneling microscopy and spectroscopy, grazing incidence X-ray diffraction

## Abstract

One-monolayer (ML) (thin) and 5-ML (thick) Si films were grown on the α-phase Si(111)√3 × √3R30°-Bi at a low substrate temperature of 200 °C. Si films have been studied in situ by reflection electron energy loss spectroscopy (REELS) and Auger electron spectroscopy, as a function of the electron beam incidence angle α and low-energy electron diffraction (LEED), as well as ex situ by grazing incidence X-ray diffraction (GIXRD). Scanning tunneling microscopy (STM), and scanning tunneling spectroscopy (STS) were also reported. The REELS spectra, taken at the Si K absorption edge (~1.840 KeV), reveal the presence of two distinct loss structures attributed to transitions 1*s*→π* and 1*s*→σ* according to their intensity dependence on α, attesting to the *sp*^2^-like hybridization of the silicon valence orbitals in both thin and thick Si films. The synthesis of a silicon allotrope on the α-phase of Si(111)√3 × √3R30°-Bi substrate was demonstrated by LEED patterns and GIXRD that discloses the presence of a Si stack of 3.099 (3) Å and a √3 × √3 unit cell of 6.474 Å, typically seen for multilayer silicene. STM and STS measurements corroborated the findings. These measurements provided a platform for the new √3 × √3R30° Si allotrope on a Si(111)√3 × √3 R30°-Bi template, paving the way for realizing topological insulator heterostructures from different two-dimensional materials, Bi and Si.

## 1. Introduction

Two-dimensional (2D) elemental materials are representatives of a family of the new allotropic structures of the groups III–VI elements of the periodic table. The existence of a 2D crystal was initially identified in carbon atoms in 2004 [1], for which the Nobel Prize in Physics was awarded in 2010 “for groundbreaking experiments regarding the two-dimensional material graphene” [2]. Graphene is a single sheet of carbon atoms arranged in a honeycomb lattice, due to its preferential *sp*^2^ hybridization atomic orbitals in a 2D sheet.

Graphene symmetry determines some of its peculiar physical properties such as electronic structures. In graphene, the electrons behave as Dirac Fermions [3], i.e., as relativistic massless particles exhibiting a linear energy band dispersion at high symmetry points K and K′ in the hexagonal Brillouin zone (BZ). As a consequence, graphene has a high electronic mobility, which makes it an ideal candidate for electronic devices, even if the tailoring of its electronic property is counteracted by the absence of gaps in K and K′ at its inequivalent Dirac points, where its linear bands meet the Fermi level.

In 2012, the first man-made archetype silicene, i.e., one-atom-thick silicon arranged in a buckled, *sp*^2^-like 2D honeycomb lattice, was synthesized by epitaxy on the (111) surface of an Ag single crystal in ultra-high vacuum (UHV) conditions from a solid Si source [4]. As a consequence of the successful synthesis of this tough material, known until its experimental creation only in its natural three-dimensional *sp*^3^ tetragonal hybridization, numerous other 2D elemental materials have been synthesized. Familiar examples, to name just a few, are borophene [5], gallenene [6], germanene [7], stanene [8], plumbene [9], phosphorene [10], arsenene [11], antimonene [12], bismuthene [13], tellurene [14], and selenene [15].

More interestingly, silicene and multilayer silicene hosted Dirac Fermions with carrier mobility, comparable to those of graphene [4,16,17,18], showing ambipolar transistor behavior [19,20], thus promoting their application in nanotechnology devices that could fit well with the actual silicon industry.

It has been widely demonstrated that, in addition to the synthesis of silicene and multilayer silicene, on a single crystal of Ag (111) [16,17,18,21,22], Ir (111) [23], and zirconium diboride [24], remarkably, they can be synthesized on Si(111), after the formation of the interface Si(111)√3 × √3 R30°-Ag [22,25,26]. This is always during the growth of the Si films, with both a constant low substrate temperature of ~200 °C and a low silicon solid source flux of ~10^−2^ ML/min, overcoming the debate about whether Ag atoms migrating onto the surface would promote the continuation of the silicon crystal, rather than the growth of buckled honeycomb layers of silicene.

Very importantly, recent measurements of angle-resolved inverse photoemission [27], by applying similar Si growth conditions, have confirmed those of angle-resolved direct photoemission performed on silicene at the Si(111) √3 × √3 R30°-Ag interface [26], and, thus, also the results from [22,25].

Reflection electron energy loss spectroscopy (REELS) and Auger electron spectroscopy (AES), low-energy electron diffraction (LEED), grazing incidence X-ray diffraction (GIXRD), scanning tunneling microscopy (STM), and scanning tunneling spectroscopy (STS) measurements are crucial to assess the physical and structural properties of silicene, unambiguously attesting to the strong relationship between the silicene atomic arrangement and its electronic structures.

Here, we demonstrate the nature of the Si chemical bonds by means of reflection electron energy loss spectroscopy, confirming the *sp*^2^-like hybridization of the silicon valence orbitals in both thin and thick silicene film, according to STM/STS, AES, LEED, and GIXRD measurements. These results are corroborated by the prediction of the silicene stability at the Si(111)√3 × √3R30°-Bi interface by density functional theory (DFT) calculations [28], leading us to verify the experimental synthesis of the growth of 1 ML of silicene and going up to a thickness of five layers.

We stress in this context that the synthesis of silicene and multilayer silicene is by no means simple and that the search for new templates for their synthesis represents a step forward for both fundamental and applied physics. In particular, the presence of a matrix composed of Bi atoms (as in the case of the α-phase Si(111)√3 × √3R30°-Bi) and silicene films can open up the possibility of new properties in the field of superconductivity, as well as in topological insulators with high spin-orbit coupling.

## 2. Materials and Methods

These experiments were performed at the CNR-ISM laboratory of Tor Vergata, Rome, Italy. The REELS and AES spectra were collected as a function of the incidence α angle between the normal surface and the primary electron beam (E_P_ = 2.5 KeV). REELS and AES data were acquired in first derivative mode with a PHY 255G double-pass cylindrical mirror analyzer equipped with a coaxial electron gun, with a resolution of at least 0.5 eV, and recorded by varying the α angle between the impinging electron beam and the normal to the sample surface, at 0°, 30°, 45°, 60°, and 75°. The Si(111) substrates (MEMC Electronic Materials, p-type B-doped, resistivity 5.5–10.5 Ω·cm) were cleaned in the UHV chamber (base pressure: 8.0–10^−11^ mbar) by several cycles of annealing at about 400 °C, to remove oxygen from the surface and after the samples were flashed at about 1150 °C. Sharp Si(111)7 × 7 LEED patterns were observed. Bi was evaporated in Si(111) kept at ~350 °C at a rate of ~0.02 ML/min from a Bi source up to ~1/3 monolayer (ML), in order to form the α-phase of Si(111)√3 × √3R30°-Bi, hereafter called Si(111)√3 × √3-Bi. Si was evaporated on top of this interface kept at ~200 °C at a rate of ~0.01 ML/min from a source of Si up to ~1 and 5 monolayers (MLs). A clear √3 × √3 Si reconstructed LEED pattern was observed. XRD were performed both in-plane and out-of-plane using a PANalytical Empyrean diffractometer (Malvern PANalytical Ltd.- Malvern, Worcestershire, United Kingdom; Almelo, Netherlands), equipped with a Cu (40 KV, 40 mA) ceramic anode, operating in Bragg–Brentano reflection mode (divergent slits). The wavelengths used were K-Alpha type: K-Alpha1 = 1.54060 (Å), and K-Alpha2 = 1.54443 (Å), with a K-A2:K-A1 ratio of 0.50000. The data acquisition was performed by a PixCel 3D solid state detector in 1D mode. The grazing incidence configuration was enhanced by applying to the sample surface a small tilt angle φ, offset of 2°, to be more surface-sensitive.

Using similar experimental setups for sample preparation, the STM and STS measurements (OMICRON LT STM/AFM) were carried out at the Institute of Physics, Maria Curie-Sklodowska University, Lublin, Poland. The α-phase of Si(111)√3 × √3R30°-Bi was obtained by postannealing for 10 min of ~0.5 ML of Bi deposited at room temperature (RT), whereas ~0.5 ML of Si was deposited on top at a rate of ~0.017 ML/min with the substrate kept at two temperatures of (240 ± 50) °C and (330 ± 50) °C. An in situ reflection high-energy electron diffraction (RHEED) pattern was used to monitor the √3 × √3 reconstructions in both Bi and Si growth, and then STM/STS measurements were performed at RT.

## 3. Results and Discussion

Here, we recall that, as a function of the growth parameters, the Bi atoms form on Si(111)7 × 7 three different structures: α-phase, β-phase, and honeycomb phase [29,30,31,32,33,34,35,36,37,38,39,40,41,42,43,44,45,46]. What distinguishes the various phases in the Si(111)√3 × √3R30°-Bi interface are the substrate temperature and the Bi coverage. The α-phase is obtained at ~340 °C with Bi coverage of 1/3 ML; β-phase appears at 250 °C and corresponds to a Bi coverage of 1 ML; indeed, the honeycomb phase corresponds to 2/3 ML of Bi obtained in the temperature range 25 °C < T < 250 °C [29,30,31,32,33,34,35,36,37,38,39,40,41,42,43,44,45,46].

After the initial studies carried out on Si(111)√3 × √3R30°-Bi interface [29,30,31,32,33,34,35,36,37,38,39,40,41,42,43,44] to date [28,45,46], great effort has been made both from a theoretical and an experimental point of view to understand the atomic arrangement of Bi atoms [28,29,30,31,32,33,34,35,36,37,38,39,40,41,42,43,44,45,46]. For α-phase Bi (1/3 ML coverage), several models have been proposed: T_4_ and H_3_ models have a threefold coordination with Si atoms from the first layer up to the second and fourth substrate layers. Indeed, the T_1_ model has the Bi atoms on top of the Si atoms of the first layer, keeping alive two Si dangling bonds; in the S_5_ model Bi atoms are substituted for the ones in the second Si layer, which takes place at the T_4_ sites. The T_4_ model is most energetically favored for the α-phase Bi [42].

The milkstool model has been proposed for β-phase (1 ML), where Bi forms trimers whose center is located up to the T_4_ site; each Bi atom forms two bonds with neighboring Bi atoms and one with the Si atom of the substrate [37,38,39].

A third phase was initially observed for 2/3 Bi coverage [43], which appears to show a hexagonal structure, but more probably is a combination of α and β phases. All three phases exhibit √3 × √3R30° surface reconstruction [28,29,30,31,32,33,34,35,36,37,38,39,40,41,42,43,44,45,46].

**Figure 1:**
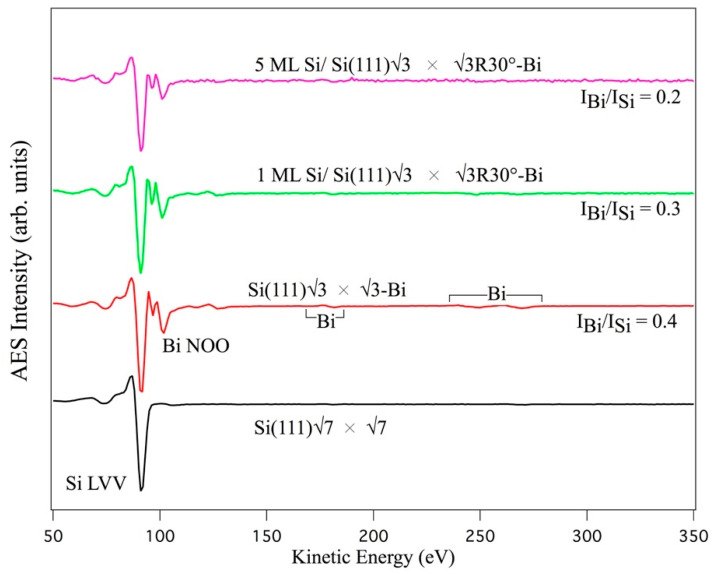
Auger spectra taken on the Si(111)7 × 7 surface reconstruction (black curve); Si(111)√3 × √3-Bi interface (red curve); 1 ML Si/Si(111)√3 × √3R30°-Bi (green curve) and 5 ML Si/ Si(111)√3 × √3R30°-Bi film (pink curve). On the right the ratios of the Si LVV and Bi NOO signals are indicated; the square brackets point to the weakest AES NNO transitions of bismuth.

Figure 1 shows the Auger spectra for the following surfaces: 7 × 7 surface reconstruction of Si(111), the α√3-Bi phase of the Si(111)√3 × √3R30 °-Bi, 1 ML, and 5 MLs of Si film on Si(111)√3 × √3-Bi, respectively, from bottom to top.

The spectrum relative to the Si(111)√3 × √3R30°-Bi interface, hereafter referred to as Si(111)√3-Bi, (red curve), collected after the formation of the interface 0.3 ML of Bi, unlike the spectrum of Si(111)7 × 7 (black curve), which shows only the AES Si LVV transition located at 91 eV, presents a new signal at 101 eV and at a higher energy of approximately 180 eV, and between 230–280 eV due to the NOO and other weaker NNO transitions of Bi.

The 1 ML √3 × √3-Si film spectrum (green curve) was obtained from Si deposition at a substrate temperature of about 200 °C. This film, on the Si(111)√3-Bi interface, again has a √3 × √3 structure (as observed by the LEED pattern reported below). The pink spectrum corresponds to about 5 MLs (four layers beyond the first) of Si deposited at the same temperature as the single layer.

As can be seen, the signal ratio (Bi NOO)/(Si LVV) decreases as the silicon deposition increases. After the deposition of the first layer of Si, the signal ratio Bi/Si decreases by 25% with respect to that of the pristine Si(111)√3-Bi interface, becoming 0.3, and becomes 0.2 after the deposition of an additional 4 MLs of Si, in line with the expected decrease in the AES signals, following the terraced Si growth of multilayer silicene on Ag (111) single crystal [18,21,22], and/or on Si(111)√3 × √3R30°-Ag [22,25,26].

Furthermore, in the spectrum related to the 1 ML Si film, the intensity of the Bi NNO transitions decreases faster with respect to the intensity of the NOO transition. In the multilayer spectrum, in pink, it is possible to observe the almost complete disappearance of the Bi NNO signals and a further decrease in the NOO.

**Figure 2:**
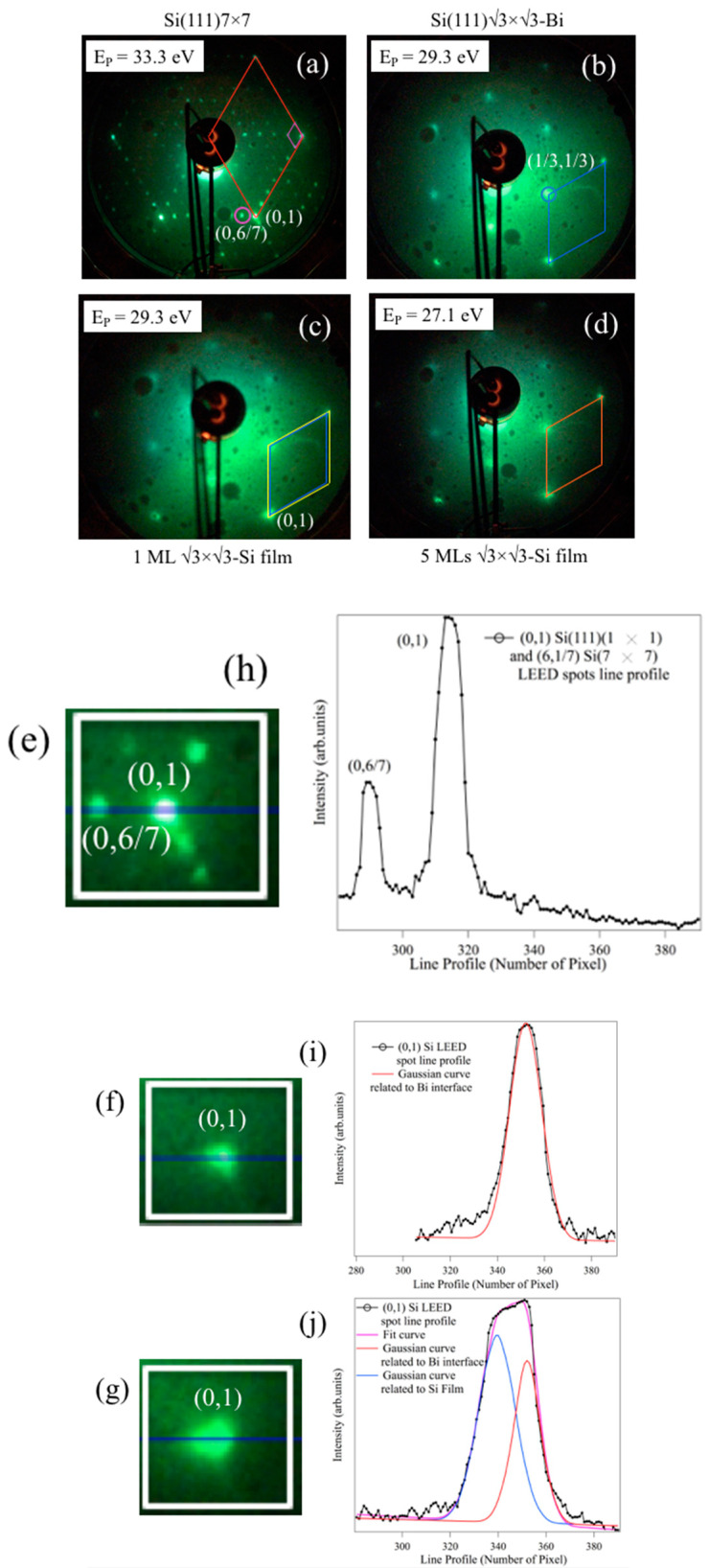
LEED patterns of the different interfaces: Si(111)7 × 7 (**a**); Si(111) √3 × √3R30°-Bi (**b**); 1 ML Si/ Si(111)√3 × √3R30°-Bi (**c**); 5 ML Si/Si(111)√3 × √3R30°-Bi (**d**). The red rhombus in (**a**) indicates the unit cell of the unreconstructed Si(111), the (0, 1) vertex is marked; the pink one instead represents the unit cell of the 7 × 7-Si surface reconstruction, one of the (6/7) spot is evidenced by the pink circle; the pattern was acquired at an energy of the primary of 33.3 eV. The pattern in (**b**) was taken at 29.3 eV, the unit cell of this structure is shown in blue, the (1/3, 1/3) spot is indicated by the blue circle; that of 1 ML of Si in (**c**) was also acquired at 29.3 eV. Here, a blue and yellow rhombus are shown, respectively, which represent the elementary unit cells of silicon on Bi and Bi on 7 × 7-Si. The patterns were recorded at the same primary energy in order to overlap them. The orange rhombus in (**d**) represents the unit cell of 5 MLs of Si. This pattern was collected at 27.1 eV. The left panels (**e**,**g**) are the magnification of both the (0, 1) and (6/7) LEED spots of the unreconstructed 1 × 1 Si(111) and reconstructed 7 × 7 Si(111) unit cells (**e**); the (0, 1) unit cell vertex of the Bi/Si(111) (**f**); and (0, 1) unit cell vertex of Si film on Bi/Si(111) (**g**) of the LEED patterns reported in (**a**–**c**). The right panels (**h**) and (**j**) are the LEED spot line profiles of (**e**,**g**). The (0, 1) LEED line profile in the Bi/Si(111) interface (**i**) is fitted with a Gaussian curve, FWHM = 15 pixels; the (0, 1) LEED line profile in Si film on Bi/Si(111), panel (**j**), is convoluted with two Gaussian curves: the blue with FWHM = 19 and the red with FWHM = 14 pixels; the pink curve is the best fit. The distance between the two Gaussian peaks is Δ^(number of pixels)^ = 12 pixels.

After the formation of each interface, the electron diffraction patterns (LEED) shown in Figure 2 were acquired.

Figure 2a shows the typical diffraction pattern of the Si(111)7 × 7 reconstructed surface. The red rhombus constitutes the unit cell Si(111)1 × 1; its vertices, located on the most intense spots, represent the integer orders, and the (0, 1) vertex is reported; the smaller pink rhombus, instead, constitutes the unit cell of the Si(111)7 × 7 reconstruction, and one of the (n/7) spots is circled. Figure 2b, obtained after the formation of the α-phase of 0.3 ML of √3 × √3-Bi on Si(111), shows the complete disappearance of its 7 × 7 reconstruction by the loss of the n/7 fractional orders (n = 1 ÷ 6), and the appearance of the new orders 1/3 related to the reconstruction √3 × √3 of bismuth, the (1/3, 1/3) spot is circled. The unit cell of this structure, represented by a blue rhombus, is rotated by 30° with respect to that of the Si(111)7 × 7. It can also be noted that the LEED pattern background is slightly modified and the spots related to Si(111)1 × 1 become more faded. Figure 2c shows the LEED pattern of 1 ML of Si deposited on the √3-Bi interface and again the background increases and the pattern becomes more and more muffled.

The unit cells of the Si film are colored yellow to show the comparison with the bismuth interface in blue. They show slightly different dimensions. Figure 2d displays the pattern acquired on a further four Si monolayers deposited at the same temperature of the single layer at a primary beam energy of 27.1 eV. These layers continue to show a clear reconstruction, √3 × √3. The unit cell is shown in orange in Figure 2d and is not appreciably different from the yellow cell shown in Figure 2c.

The comparison between the unit cells in Figure 2c shows that, in the reciprocal space, the unit cell relative to 1 ML of Si (yellow rhombus) is larger than that of bismuth (blue rhombus). The energy of the primary beam for the two patterns (Figure 2b,c) was kept constant at 29.3 eV, thus making possible a direct comparison between the dimensions of the lattice parameters of the two structures. The silicon atoms rest on top of the bismuth interface, maintaining the same √3 × √3 reconstruction and exhibiting a side of the unit cell greater than that of bismuth, in the reciprocal space.

To evaluate a possible expansion of the unit cell in the reciprocal space of the √3 × √3 Si film, compared to that of Bi, it is proposed that the (0, 1) LEED spots of the Figure 2a–c panels be scrutinized by analyzing their LEED line profiles.

In the panels of Figure 2e–g is displayed the magnification of the (0, 1) integer order of the unreconstructed 1 × 1 Si(111) unit cell vertex, and the fractional (6/7) LEED spots of the 7 × 7 Si(111) reconstruction of Figure 2e; the (0, 1) unit cell vertex of the Bi/Si(111) interface, and that of Si film on Bi/Si(111) are marked in Figure 2f,g of the LEED pattern shown in Figure 2a–c. On the other hand, in the right panels (Figure 2h–j) are shown the LEED spot line profiles of the LEED spots (Figure 2e–g).

The (0, 1) integer and fractional (6/7) order LEED spots of Si(111) and their reconstruction in Figure 2e are sharp and well defined, as evidenced by the line profile in Figure 2i. This line profile details both spots, giving to the (0, 1) integer order a width of 8 pixels.

The (0, 1) LEED line profile at the Bi/Si(111) interface of Figure 2i is overlapped to a Gaussian curve (the continuous red curve with FWHM = 15 pixels), denoting a good match between the unit cell of √3 × √3 Bi and that of the Si surface. The (0, 1) LEED line profile in the Si film on Bi/Si(111) (Figure 2j) is convoluted with two Gaussian curves: the blue with FWHM = 19 pixels and the red with FWHM = 14; the green curve is the best fit. The distance between the two Gaussian peaks is Δ^(number of pixels)^ = 12 pixels.

These results obtained by LEED analysis are extremely important as they assume an expansion of the Si film mesh compared to that of bismuth. This expansion, using the distance between the two Gaussian peaks, corresponds to 3.5%:

(1) $$\frac{a_{\sqrt{3}}^{*\;Si\;film}-a_{\sqrt{3}}^{*\;Bi}}{a_{\sqrt{3}}^{*\;Si\;film}}\;\sim \;0.035.$$

Considering that, in real space, the side of the unit cell √3 of bismuth is $a_{\sqrt{3}}^{Bi}=a_{\sqrt{3}}^{Si\;sub}=6.65$ Å, it can be determined that the side of Si√3, which we call $a_{\sqrt{3}}^{Si\;film}$, is ~6.42 Å, then smaller than $a_{\sqrt{3}}^{Si\;sub}$. Therefore, according to the analysis of LEED patterns, the film Si √3 × √3 has a contracted mesh of about 3.5%:

(2) $$\frac{a_{\sqrt{3}}^{\;Si\;sub}-a_{\sqrt{3}}^{\;Si\;film}}{a_{\sqrt{3}}^{\;Si\;sub}}\;\sim \;3.5\%.$$

This is an important result and is in agreement with what has been obtained for the growth of Si on the interface Si(111)√3 × √3-Ag [22,25,26].

**Figure 3:**
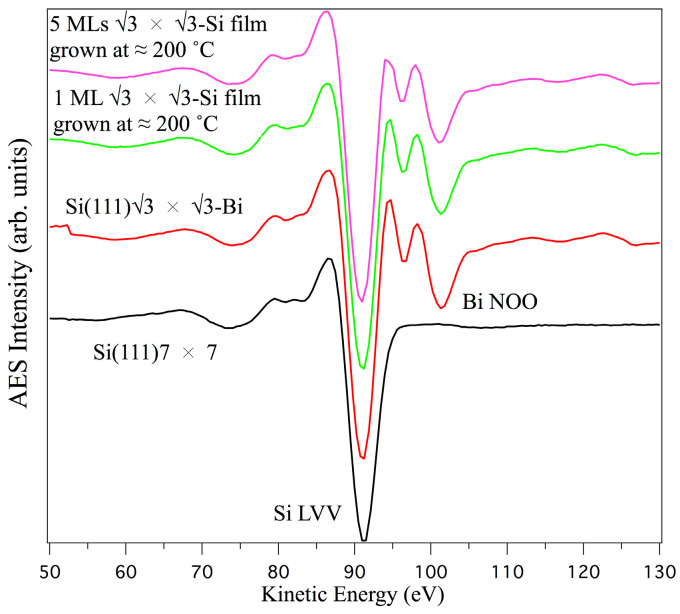
Si LVV line shape of: Si(111)7 × 7 (in black); Si(111)√3 × √3-Bi (in red); 1 ML Si/Si(111)√3 × √3-Bi (in green); 5 ML Si/Si(111)√3 × √3-Bi (in pink).

Figure 3 shows the Auger line shape of the Si(111)7 × 7 interfaces (in black); Si(111) √3 × √3-Bi (red curve); 1 ML Si/Si(111)√3 × √3-Bi (green curve); 5 ML Si/Si(111)√3 × √3-Bi (purple curve), at α = 0°, to better asses the crucial changes present in the NOO transition of bismuth and LVV of silicon.

**Figure 4:**
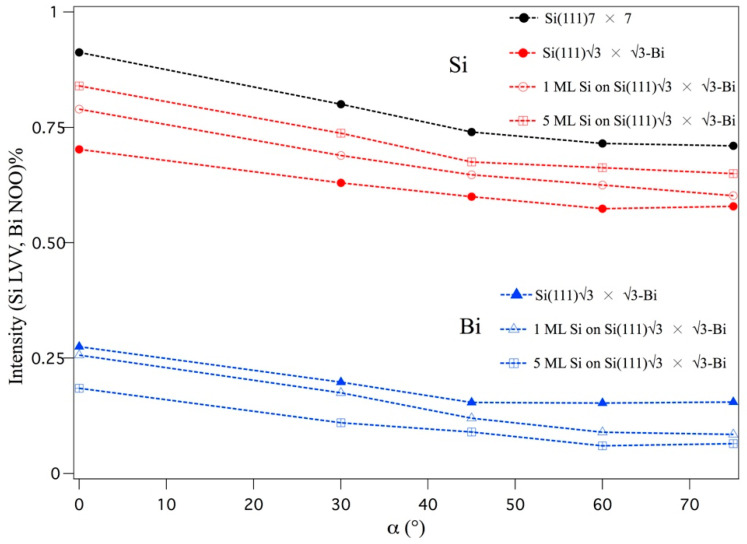
Peak-to-peak intensity of the Bi NOO and Si LVV transitions as a function of angle α, the angle between the impinging electron beam, and the normal to the sample surface, for the different interfaces. The dashed lines are a guide for the eyes.

The spectra shown in Figure 1 and Figure 3 were acquired not only for α = 0° (α is the angle between the impinging electron beam and the normal to the sample surface) but also for values of α of 30°, 45°, 60°, and 75°, to determine the curves plotted in Figure 4, where PPI (the peak-to-peak intensity of the Bi NOO and Si LVV transitions) is plotted as a function of angle α. The solid lines are only a guide to distinguish the various points. From this graph, we get three important pieces of information: the curves follow the same trend as the angle α increases; the intensity of the bismuth signals decreases as the silicon deposit increases; and the silicon signal increases.

From an inspection of the curve behavior of Figure 4, it is evident that the AES intensities, relating to the two elements Bi and Si, have been reduced by a factor of 0.3. This initially corresponds to the formation, at α = 0°, of the Si–Bi interface.

Each point that determines the decreasing trends of Figure 4, for both Si and Bi atoms in the formation of the various interfaces, contains the intrinsic contribution of the experimental setup and is derived from the growth mode of the Bi interface and Si films.

Bear in mind that the electron analyzer (CMA) has a collecting angle of γ = 42.3° on its annulus over the entire solid angle. Varying the angle α changes at the same time the angle of emission of the electrons from the sample that come both from the surface and bulk layers, and, in part, the contribution to the reduction of signal intensities, also due to the loss of an active part of the electron analyzer, related to the geometry of the CMA. Note that this intrinsic contribution, linked to geometry, is the same for both Si and Bi. As a consequence, going into detail on the trends in Figure 4, the fact that the curves of the Si films are always positioned above those measured after the formation of the Bi–Si interface is in line with the hypothesis that the Bi atoms are allocated on the surface of the Si(111) substrate and that the growth of Si proceeds on a subsequent layer on top of the previous one.

This Si growth can be defined as terraces mode, in analogy to those of multilayer silicene grown on Ag(111) and/or Si(111) after the Si-√3 interface formation [18,21,22,25,26], due to the incomplete disappearance of Bi signals after the five-layer deposition of Si.

REELS measurements, which provide the hybridization character of the Si–Si bonds, are used here to establish silicon growth on Si(111)√3 × √3 R30°-Bi. First of all, we have to keep in mind that in an electron energy loss process there is the breakdown of the dipole selection rule (∆l = ±1) [47], which usually controls the photon absorption of the electronic transitions from a core level, initial state, to empty final states. In this case, the two orbitals’ final states with π* and σ* characteristics are available for transitions from the Si initial state *s*, the Si K threshold. These measurements were crucial in assigning the behavior of the quasiplanar valence bonds in synthesized silicon allotrope, establishing a fingerprint of the *sp^2^*-like configuration in silicon nanoribbons (SiNRs) [48].

**Figure 5:**
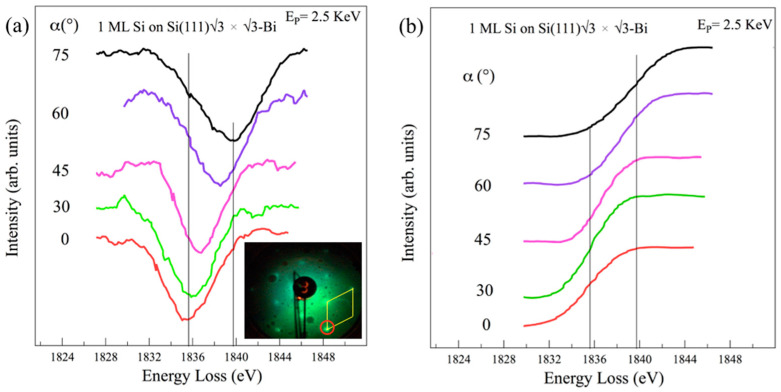
REELS spectra at the Si K edge from the 1 ML of Si grown on Si(111)√3 × √3-Bi interface collected in AES mode (**a**) and numerically integrated (**b**), going from normal (α = 0°) to almost grazing (α = 75°) incidence. Energy loss spectra refer to the primary elastic peak E_P_ = 2.5 KeV. The LEED pattern shown in (**a**) was collected at a primary energy of 29.3 eV; red circle: silicon integer order spot; yellow rhombus is the √3 × √3 silicene reconstruction.

Figure 5 displays the REELS spectra at the K Si absorption edge from the 1 ML √3-Si grown on the Si(111)√3-Bi interface measured in derivative mode (Figure 5a) and numerically integrated (Figure 5b). Spectra were acquired at various angles of incidence of the primary electron beam, from α = 0° (parallel) to 75° (almost perpendicular) to the normal surface.

The spectrum collected at α = 0° in Figure 5a shows a Si loss located at 1835.6 ± 0.5 eV. As the angle α varies, the intensity of this structure is reduced and compensated for by the presence of a new structure a (1839.7 ± 0.5) eV that becomes more and more prominent as the angle of incidence increases (α = 75°). These two structures indicate the presence of two different transitions that start from the Si K (1*s*) initial (filled) state of the silicon to arrive at the first two empty states located beyond the Fermi level. The two loss structures have been attributed to the excited transitions 1*s*→π* normal incidence and 1*s*→σ* grazing incidence, giving that the silicon atomic valence orbitals have s and p characteristics. At α angles between 30° and 60°, the two structures can persist at the same time as they are excited by the parallel *s* and perpendicular *p* components of the momentum electron beam projection. In electron energy loss spectroscopy, the exchanged momentum, indeed, is related to the polarization vector in optics.

The integrated spectra of the loss structures of Figure 5a are shown in Figure 5b. An energy loss difference ∆E of 4.1 ± 0.5 eV is clear when the Si K absorption threshold passes from α = 0° to 75°; if at intermediate angles, it is difficult to sharply separate the two transitions, mainly due to poor energy resolution or a low-intensity signal. Thus, the continuous shift of the Si K edge, by varying the incidence α angle, is in line with the increased intensity of the 1*s*→σ*, while the other 1*s*→π*disappears.

Both this behavior and the energy difference between the two loss structures can be identified by the mixed hybridization in between *sp*^2^ and *sp*^3^, with the sp*-like character of the Si–Si bond being in agreement with what has already been observed for silicene nanoribbons [48].

**Figure 6:**
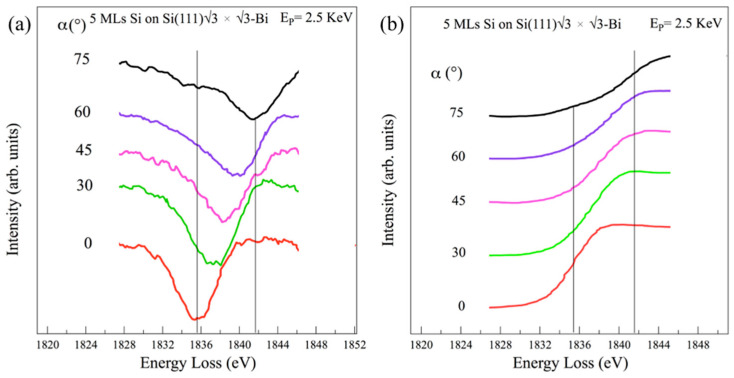
REELS spectra of the Si K threshold at 5 MLs silicon deposition as a function of the α angle primary beam incidence, acquired in AES mode in (**a**) and numerically integrated in (**b**). The spectra refer to the elastic peak of the primary, E_P_ = 2.5 KeV. The spectra are normalized at α = 0°.

For 5 MLs of silicon deposition, the loss structure present at α = 0° in Figure 6a is always found at an energy of 1835.6 ± 0.5 eV. As the angle of incidence α increases, the intensity of this structure is attenuated, while a different structure located at 1841.8 ± 0.5 eV takes place, becoming the most intense.

In the integrated spectra of Figure 6b, the absorption Si K edge from α = 0° to α = 75° shows a shift with an energy difference of ∆ = 6.2 ± 0.7 eV, which could predict the more *sp*^2^-like character of the silicon multilayer, in comparison with the findings obtained for carbon. Indeed, these results are very close to those obtained for K thresholds of graphite and graphene [47,49,50,51], or hexagonal boron nitride [52], where a comparable angular dependence of the 1*s*→π* and 1*s*→σ* transitions of their *sp*^2^ orbitals was found, although more quantitative DFT calculations, for both a buckled single layer and multilayer silicene, in combination with the charge transfer from the two expected sublattices, could give relatively pure and/or mixed *sp*^2^/*sp*^2^-*sp*^3^ configurations.

**Figure 7:**
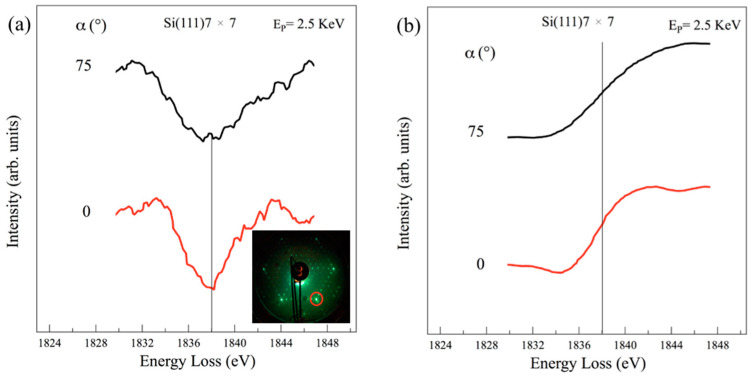
REELS spectra at the Si K edge from the Si(111)7 × 7 collected in derivative mode (**a**) and numerically integrated (**b**), at normal (α = 0°) and grazing (α = 75°) incidence. The energy loss spectra refer to the primary elastic peak E_P_ = 2.5 KeV. There is no shift of either the loss feature or the edge. The LEED pattern shown in (**a**) was collected at primary energy of 33.3 eV; the red circle is the silicon integer order spot.

For comparison, Figure 7 shows the Si K edge from the Si(111)7 × 7 surface reconstruction, with Figure 7a,b being the derivative and its numerically integrated loss spectra. Here, we can observe the presence of a single loss structure at 1838.0 ± 0.5 eV, both at α = 0° and α = 75°. This is associated with the unique absorption transition 1*s*→σ*π* of the isotropic *sp*^3^ orbitals in the cubic structure of diamond-like silicon, where, as expected, no angular dependence is given.

Therefore, the presence of the two transitions/edges at the Si K threshold and their angular dependences in 1 ML and 5 MLs Si deposition on the Si(111)√3 × √3-Bi interface could be the fingerprints of the *sp*^2^-like hybridization of the Si valence electrons within the silicon layers. This is a crucial result that can demonstrate a unique honeycomb-like structure adopted by the silicon atoms when organized as a √3 × √3 reconstruction on the Si(111)√3 × √3-Bi template.

**Figure 8:**
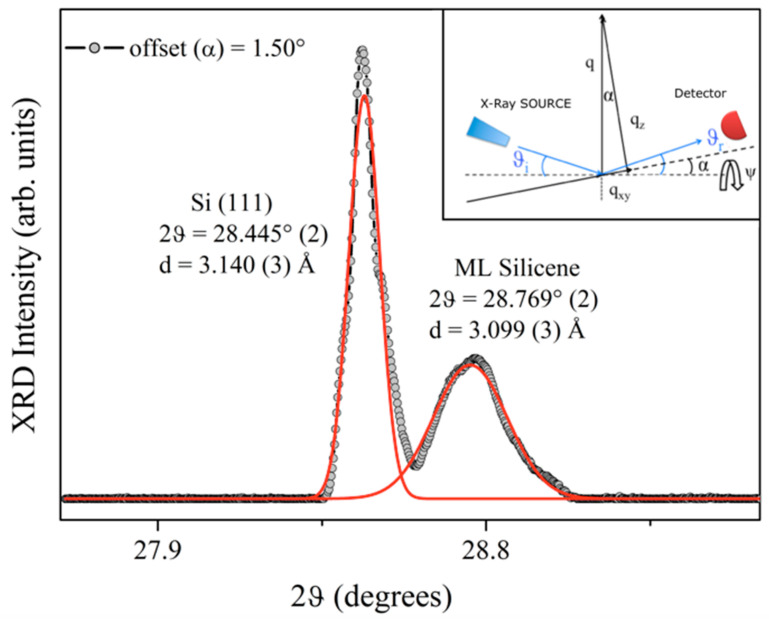
XRD pattern (gray dots) collected at an offset α =1.50° and ψ = 0.0° from 5 MLs of Si on Si(111)√3 × √3-Bi interface. Distances of planes in the Si(111) substrate and 5 Si ML film are reported as deduced by the Gaussian fit (red lines) of the XRD peaks (FWHM Si = 0.093°; FWHM silicene = 0.227°. In the inset, the schematization of the experimental setup is reported, allowing for the momentum transfer to also be sensitive to the surface structure: θ_i_ = θ_r_, the incident and reflected scattering angles; α is the in-plane tilt; ψ is the out-of-plane tilt.

Now we take into consideration the XRD measurements shown in Figure 8. They are fundamental to simultaneously validate the side of the √3-Si elementary mesh, as well as the average distance between the planes perpendicular to the sample surface.

Out-of-plane X-ray diffraction patterns were collected and, subsequently, a rocking curve (RC) was performed in order to minimize the substrate silicon contribution. In Figure 8, the XRD pattern collected by tilting the sample at α = 1.00° is reported and the schematization of the experimental setup is shown as an inset. By tilting the sample along the α-direction, i.e., the X-ray plane, the whole scattering angle is preserved and thus the position of the reflections is, too; however, the intensity of monocrystalline silicon is much reduced, thus allowing for accurate and very resolved detection of contributions close to the dominant substrate signal. Indeed, from the X-ray diffraction pattern presented in Figure 8 collected upon the 5 ML Si film, we can, interestingly, observe the presence of two distinct peaks located at the angles of 2θ = 28.445° (2) and 2θ = 28.769° (2), as in the case of √3 × √3-Si on Ag(111) single crystal [21,22], and √3 × √3-Si on Si(111)- √3 × √3-Ag [25,26].

The first peak is attributed to the (111) reflection of the {111} planes of the silicon substrate. The second peak corresponds to the diffraction between the planes of the Si film multilayer. Applying Bragg’s law, n λ = 2d• sin θ, where λ is the wavelength of the incident radiation λ (K_α_^Cu^ = 1.540 Å), and n = 1 is the order of the reflection, we obtain d_(Si(111))_ = 3.140 Å (3) and d_(Si film)_ = 3.099 Å (3), in good agreement with the values obtained in [21,22,25,26].

**Figure 9:**
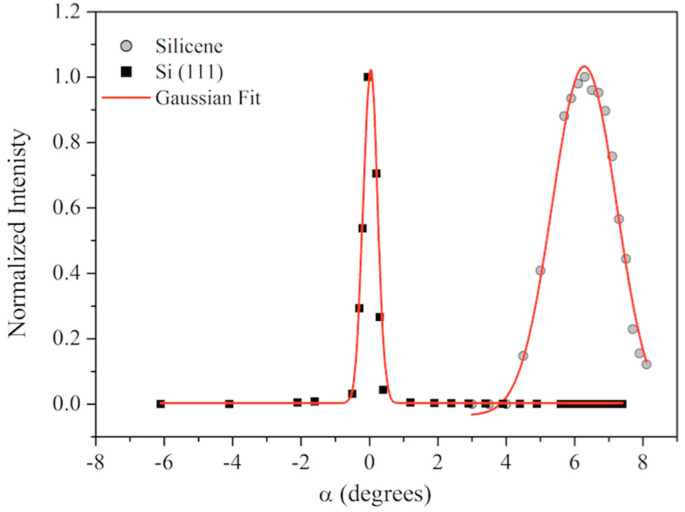
Rocking curves of the Si(111) (black squares) and Silicene 5 ML (gray dots) out-of-plane reflections. The Gaussian fit (red lines) of each RC is reported: FWHM Si = 0.400°; FWHM Silicene = 2.200°.

Figure 9 gives the RC analysis, with the normalized intensities of the Si(111) and silicene contribution plotted as a function of the tilt angle α. The intensity distribution of the silicene ML and Si substrate is different, accounting for a larger mosaic spread of the silicene film with respect to the monocrystalline Si(111) substrate.

Indeed, the epitaxy index, estimated as the FWHM of the Gaussian fit of each RC reported in Figure 9, is approximately 2.200 ± 0.005° for silicene and 0.400 ± 0.005° for the Si substrate.

Furthermore, a 6.3° miscut is detected due to the Si(111)√3 × √3-Bi interface acting as a template.

**Figure 10:**
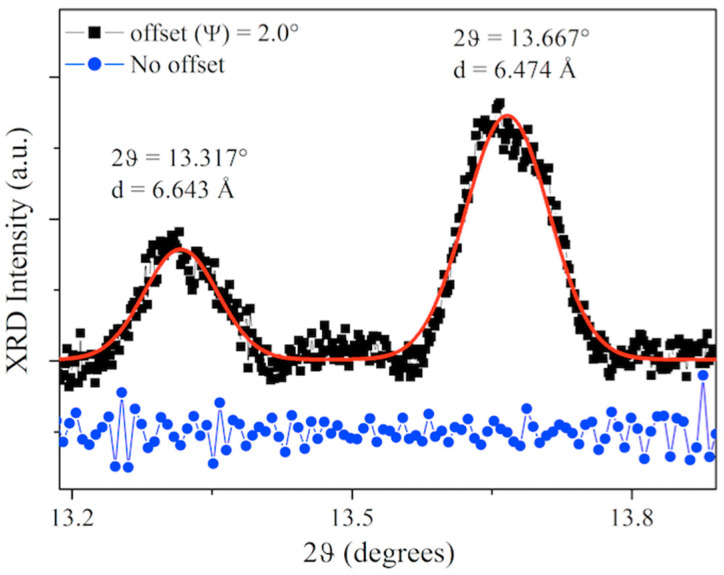
(GIXRD pattern (black squares) collected from 5 MLs of Si on Si(111)√3 × √3-Bi interface (black line, offset of ψ = 2.0° offset) compared to the XRD pattern collected without offset (blue dots). Distances of in-plane lattice sizes for the Si(111) substrate and the 5 Si MLs film are reported as deduced by the Gaussian fit (red lines) of the GIXRD peaks. FWHM (d = 6.643 Å) = 0.087°; FWHM (d = 6.474 Å) = 0.099°.

Subsequently, in order to detect the in-plane lattice parameters, samples were tilted along the ψ out-of-plane tilt, thus transferring part of the X-ray momentum transfer along the surface. In this way, the GIXRD measurements were performed; the results are presented in Figure 10. It is clear that, when the collected momentum is along the z-direction only (no tilt—blue line), no in-plane diffraction signal is observed. However, when ψ = 2.0° is applied, in-plane reflections at 2θ = 13.317° (2) and 2θ = 13.667° (2) were observed, corresponding to interplanar distances of d = 6.643 Å and d = 6.474 Å, respectively.

In fact, contraction of the unit cell side of the √3- Si film and of the mean distance of the Si film planes was detected by GIXRD measurements. The results show, at first impact, that the silicon film is structurally organized in a different way with respect to conventional tetragonal Si, similar to what was obtained for the single and multilayer silicene grown on Si(111)√3 × √3-Ag [25,26] and Ag (111) single crystal [21,22].

It is worth noting that these results are in good agreement with the DFT calculations [28] on the formation of one honeycomb buckled silicon layer on the phase α of Si(111)√3 × √3-Bi, which predicted the possibility of obtaining silicene with a distance from the Si–Bi interface of ~3.034 Å [28]. Based on the XRD structural results, we argue for the synthesis of a silicene monolayer and multilayer silicene on the Si(111)√3 × √3-Bi α-phase template. similar to what was found in [21,22,25,26], and in agreement with the set of results obtained here on *sp*^2^–*sp*^3^ hybridization for single-layer and a more planar *sp*^2^-like Si stacked film.

We are now going to scrutinize the structural and electronic properties obtained through the STM and STS measurements.

**Figure 11:**
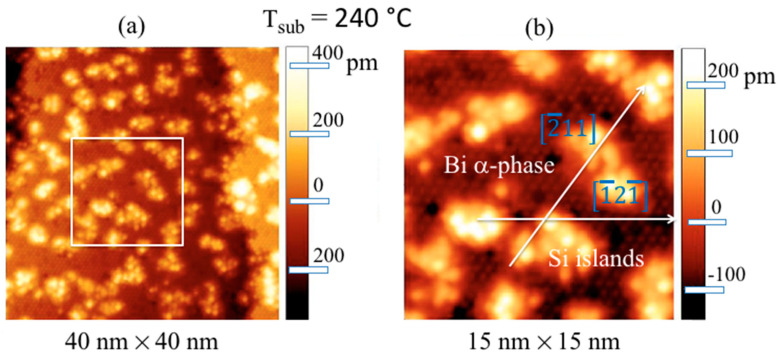
Filled-states STM images with a sample bias of −2.5 V, I = 50 pA from 0.5 ML of Si deposited at 240 °C in Si(111)√3 × √3-Bi α-phase (40 × 40) nm^2^ (**a**); zoom-in (15 × 15) nm^2^ (**b**) of the region within the white square of (**a**). The Bi α-phase and Si islands in (**b**) are marked, and the two √3 × √3- Bi lattice vector directions are labeled with white arrows.

Figure 11 displays the STM images (40 × 40) nm^2^ collected on 0.5 ML of Si deposited at 240 °C in Si(111)√3 × √3-Bi α-phase (Figure 11a) and the (15 × 15) nm^2^ zoom-in (Figure 11b) of the region within the white square of Figure 11a. The scattered bright Si islands with a lateral average side of ~3 nm, clearly evident in Figure 11a, sit on top of the dark continuous Bi α-phase regions, covering two underlying Si terraces whose appearance is bias-independent [46]. Although it is difficult to visualize their atomic configuration, in Figure 11b it does not escape the eye that they are massively aligned along the two preferential directions $\left[\bar{2}11\right]\;$ and $\left[\bar{1}2\bar{1}\right]\;$ of the atomic arrangement of the Bi atoms located below.

**Figure 12:**
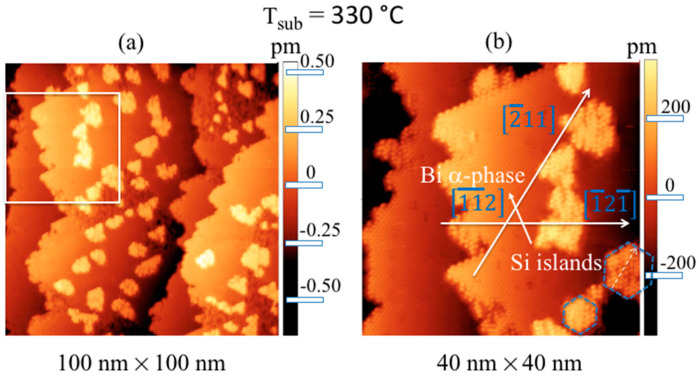
Filled-states STM images at a sample bias of −2.5 V, I = 50 pA from 0. 5 ML of Si deposited at 330 °C on Si(111)√3 × √3- Bi α-phase (100 × 100) nm^2^ (**a**); zoomed-in (40 × 40) nm^2^ view (**b**) of the region within the white square of (**a**). The Bi α-phase and Si islands in (**b**) are marked, as well as the main crystallographic directions, $\left[\bar{2}11\right]$, $\left[\bar{1}2\bar{1}\right]$, and $\left[\bar{1}\bar{1}2\right]$, of the Bi interface, as labeled with arrows, creating a hexagon. The dashed white arrow in the right corner of the image on a Si island is highlighted. The two blue dashed hexagons are rotated 30° from the main direction $\left[\bar{2}11\right]$, highlight the Si islands’ shape.

By increasing the substrate temperature during the deposition of 0.5 ML Si over the Bi α-phase, more ordered Si islands with a larger lateral size (ranging from ~9 to 20 nm) than those obtained at a temperature of 240 °C are obtained. This can be easily observed in the filled-states STM image shown in Figure 12a, where the terraces of Si are entirely covered by a very ordered α-phase of Bi, and the silicon islands appear to be atomically well organized. This can easily be observed in Figure 12b, which shows a higher-resolution STM image of the region comprised within the white rectangle in Figure 12a. Here, the three main crystallographic directions, $\left[\bar{2}11\right]\;$, $\left[\bar{1}2\bar{1}\right]\;$, and $\left[\bar{1}\bar{1}2\right]\;$, are marked, that denotes the texture of the √3 × √3 Bi reconstruction. Interestingly, the arrows include a direct continuation of the Si atoms’ arrangement, highlighting the presence of the √3 × √3 reconstruction on all Si island surfaces. The dashed white arrow, parallel to the $\left[\bar{2}11\right]$ direction, on the right corner of Figure 12b confirms the common alignment of these Si islands. Although these STM images are atomically resolved, it is far from easy to directly visualize the lattice of the silicon islands. Despite everything, intriguingly enough, it is possible to observe that most of the islands have a characteristic shape, shown in Figure 12a by two dashed hexagons rotated 30° from the main direction $\left[\bar{2}11\right]$, as a guide for the eyes; these derive from the repetition of the elementary cells of the honeycomb arrangement attributed to the Si overlayer, leaving the Si–Bi boundaries almost undisturbed.

**Figure 13:**
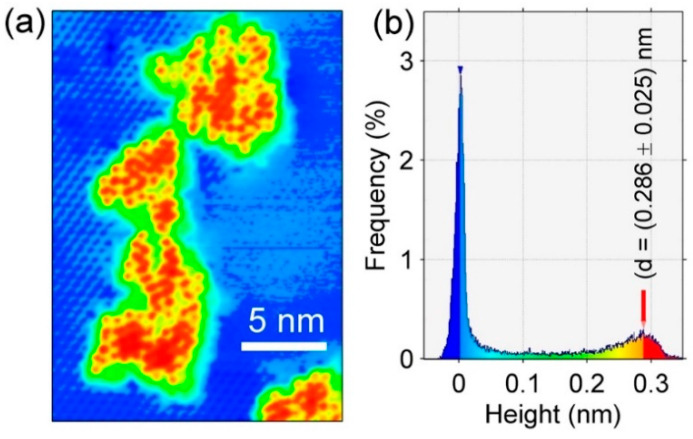
False-color STM image of Bi α-phase (blue/light blue) and Si islands (red/yellow) (**a**); height histogram of Si islands (**b**). The height difference is 0.286 ± 0.025 nm.

Figure 13a reports the STM image in false color of Bi α-phase (blue/light blue) and Si islands (red/yellow) included in the white rectangle of Figure 12a, while Figure 12b displays the height of these silicon pitches. Their height, as evidenced in the histogram of Figure 13b and probed on numerous islands, is 0.286 ± 0.025 nm, i.e., much less than a bilayer of Si(111), (0.312 ± 0.020) nm.

The height of these Si islands is just less than the value provided here by the XRD measurements on 5 MLs of Si grown on Bi α-phase, which is in line with that obtained on single-layer [4] and multilayer silicene on its 3 × 3 reconstruction on Ag (111) single crystal [18,21,22,25,26], with DTF calculations [28] and, very importantly, different from the distance of two (111) planes of silicon in its own tetragonal structure, highlighting the compliance with the assumption of having obtained the silicene phase on the α-phase √3 × √3 Bi interface. This is in agreement with previous AES, REELS, LEED, and XRD spectroscopic and structural measurements previous reported, which confirmed the synthesis in the √3 × √3 Bi α-phase on Si(111) of a single and multilayer layer *sp*^2^-like Si, called here, for simplicity’s sake, silicene and multilayer silicene. Interestingly, we noted that, in the case of Si grown in Bi α-phase, silicene can be obtained at temperatures up to 330 °C, unlike what was obtained on Ag (111), where the substrate temperature was extremely critical, around 200 °C, for the successful acquisition of silicene and multilayer silicene, instead of ordinary silicon [18,21,22,25,26].

**Figure 14:**
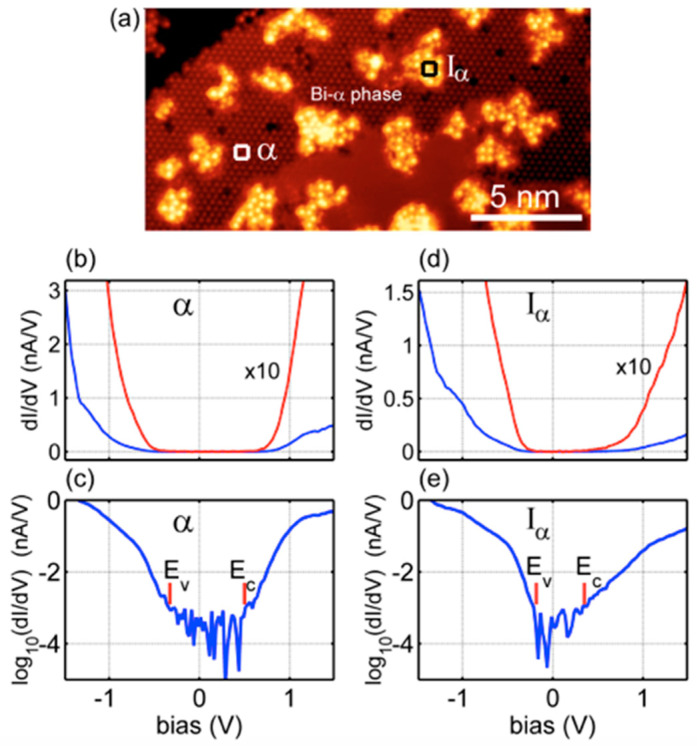
Filled-states STM image at a sample bias of −2.5 V, I = 500 pA from 0.5 ML of Si deposited at 240 °C in Si(111)√3 × √3- Bi α-phase (10 × 20) nm^2^ (**a**). Squares α and I_α_ mark the points where the STS was collected. dI/dV (nA/V) and log_10_(dI/dV) (nA/V) behavior collected in α point for Bi α-phase (**b**) and (**c**); dI/dV (nA/V) and log_10_(dI/dV) (nA/V) behavior collected at I_α_ point for Si islands (**d**) and (**e**). The red curves are magnified 10 times from the blue curves.

The STS measurements are presented in Figure 14b–e. Figure 14b,d shows the numerically calculated dI/dV characteristics as blue and red curves, where the red ones are 10 times magnified, collected on α and Iα points, i.e., Bi α-phase and Si islands. Figure 14c,e show the data as log_10_(dI/dV) curves for both points, which was necessary because the dI/dV characteristics rapidly approach the zero of dI/dV, making energy gap determination very difficult and not reliable. With the logarithm of dI/dV, one can easily define the bias range where tunneling current noise, as low as a few tenths of fA, dominates—that is, the energy range where the density of states is negligible, as expected for the energy gap E_g_. Thus, the E_g_ and the Fermi level E_F_ position above the valence band edge E_V_ from the log_10_(|dI/dV|) STS curves can be determined on Bi α-phase and Si islands, giving values of E_g_^α-phase^ = (0.91 ± 0.06) eV, E_g_^Si Islands^ = (0.53 ± 0.04) eV; and E_F_^α-phase^ = (0.33 ± 0.04) eV, and E_F_^Si Islands^ = (0.18 ± 0.04) eV. It is worth mentioning that recent DFT calculations [46] predicted for Bi α-phase an indirect E_g_ of ~0.9 eV and ~0.75 eV [28], and that the Fermi level, E_F_^Si Islands^, is shifted from 0.33 eV to 0.18 eV, above the edge of the valence band. The STM data indicate that the crystallinity of Si island is not perfect; therefore, one can expect that it may be a source of localized surface state. The strongly enhanced density of states above E_F_ seen in Figure 14e seems to confirm such a scenario.

## 4. Conclusions

To summarize, we reported the epitaxial growth of Si films on the reconstructed Si(111)7 × 7 surface passivated by 0.3 ML of Bi: the α-phase Si(111)√3 × √3-Bi. LEED, AES, REELS, XRD, STM, and STS were applied to investigate these interfaces.

These results evidenced that the √3 × √3-Si, after the Si deposition on the Bi α-phase, has a larger unit cell in the reciprocal space (by 3.5%) than that of Si(111)√3 × √3-Bi (and of the Si √3 × √3 unreconstructed substrate). A mixed *sp*^2^/*sp*^3^ hybridization for the first Si deposited layer was found, while there was a more sp^2^-like hybridization (expected to be more planar, but probably still buckled) for the 5 MLs Si film,. A new peak was obtained, distinct from that of the reflection (111) of the silicon, relative to the diffraction of a smaller distance between the planes of the Si film. The unit cell size of √3 × √3-Bi and √3 × √3-Si was 6.643 Å and 6.474 Å, respectively, as measured by grazing incidence XRD on 5 MLs Si film, pointing to a Si film with a structure similar to that obtained for multilayer silicene on Si(111)√3 × √3-Ag [26] and confirming the DFT calculations [28]. From STS, the Fermi level, E_F_^Si Islands^, was found to be shifted up from 0.33 eV (Bi α -phase) to 0.18 eV (Si islands), above the valence band edge. In addition, both XRD and STM measurements calibrate the Si stack of 0.3099 (3) nm, from XRD in the multilayer of silicon deposition, and of d_Si-Bi_ = (0.286 ± 0.025) nm, from STM imaging on Si islands, excluding the formation of ordinary Si(111), whose stack height was found to be 0.3140 nm (3) and (0.312 ± 0.020) nm, respectively, and promoting the synthesis of silicene and multilayer silicene on the α-phase Si(111)√3 × √3R30°-Bi.

## Data Availability

The data presented in this study are available upon request from the corresponding authors.
